# A consequence of mass incarceration: county-level association between jail incarceration rates and poor mental health days

**DOI:** 10.1186/s40352-022-00194-6

**Published:** 2022-10-21

**Authors:** Ashley Hickson, Ritika Purbey, Lorraine Dean, Joseph J. Gallo, Roland J. Thorpe, Keshia Pollack Porter, Aruna Chandran

**Affiliations:** 1 Fort Worth, USA; 2 Public Health, Johns Hopkins Bloomberg School of Public Health, Baltimore, MD USA; 3 Department of Epidemiology, Johns Hopkins Bloomberg School of Public Health, 615 N. Wolfe Street, Room E6650, Baltimore, MD USA; 4 Department of Mental Health, Johns Hopkins Bloomberg School of Public Health, 624 N. Broadway, Hampton House 792, Baltimore, MD USA; 5 Department of Health, Behavior, and Society, Johns Hopkins Bloomberg School of Public Health, 624 N. Broadway, Hampton House 708, Baltimore, MD USA; 6 Department of Health Policy and Management, Johns Hopkins Bloomberg School of Public Health, 624 N. Broadway, Hampton House 380A, Baltimore, MD USA; 7 Department of Epidemiology, Johns Hopkins Bloomberg School of Public Health, 615 N. Wolfe Street, Room W6501, Baltimore, MD USA

**Keywords:** Mental health, Mass incarceration, Community health, County jail, Public health

## Abstract

**Introduction:**

Mass incarceration has mental health consequences on those directly affected; some studies have also shown spillover effects on the physical health of the surrounding population. There is a dearth of research on the spillover mental health consequences of mass incarceration. This study aimed to quantify a consequence of mass incarceration which may adversely affect the population’s health and widen health disparities.

**Methods:**

Using data from the Vera Institute’s *Incarceration Trends 2.2* and the Robert Wood Johnson County Health Rankings, the association between county-level (*n* = 2823) counts of jail incarceration and reported number of poor mental health days within the past 30 days in the United States in 2018 was examined. To conduct the analysis, a negative binomial regression model was fit, adjusting for State and key demographic covariates.

**Results:**

A change in jail incarceration rate from the first to the second and third tertiles was associated with 10.14% and 14.52% increases, respectively. For every 1% increase in the rate of mass incarceration, there was a statistically significant 15% increase in the average number of reported poor mental health days over the past 30 days.

**Discussion:**

Mass incarceration is a threat to mental health as well as the well-being of the surrounding population. This can be attributed to the spillover effects that extend beyond those who are directly affected by mass incarceration.

Interventions to reduce jail incarceration as well as address the mental health needs of those living in high-incarceration rate areas should be prioritized in order to reduce health inequities and augment health outcomes for all residents of the United States.

## Introduction

Local jails have been credited as the “front door of mass incarceration” in the United States (Incarceration’s front door | Vera Institute, n.d.). Of the 2.3 million people that are incarcerated, approximately 23% are confined in local jails; the local jail population is second only to the state prison population and is nearly three times the size of the federal prison population (Mass incarceration: The whole pie 2020 | Prison Policy Initiative, n.d.).

According to the Bureau of Justice Statistics, jails are intended to be short-term facilities that confine people awaiting trial or sentencing or both, and people sentenced to a year or less (Frequently asked questions | Bureau of Justice Statistics, n.d.). Jails have the historical purpose of detaining people who were awaiting trial or sentencing and were threats to the public or at risk of fleeing; instead, they have become massive warehouses largely for people too poor to post bail or too sick for community resources to help (Incarceration’s front door | Vera Institute, n.d.). The consequences of local confinement extend beyond the jail cell and impact entire communities. Within communities, high rates of incarceration disrupt social and family networks, reduce the community’s economic potential, and perpetuate distrust and resentment towards law enforcement (Stemen, 2017).

Wildeman and Wang (2017) asserted that excessive incarceration could harm entire communities and might partly underlie health disparities both within the United States and between the United States and other countries (Wildeman & Wang, 2017). Nationally, African Americans are incarcerated locally at almost four times the rate of white Americans, despite comprising only 13% of the population (Incarceration’s front door | Vera Institute, n.d.). Within specific localities, inequities can be even more pronounced: in New York City, for example, African Americans are jailed at nearly 12 times and Latinos more than five times the rate of whites (Incarceration’s front door | Vera Institute, n.d.). In 2017, Nowotny and Kuptsevych-Timmer called for the reframing of mass incarceration as a social determinant of health, positing that incarceration should be considered in any discussion of health disparities given its negative implications for population health (Nowotny & Kuptsevych-Timmer, 2018).

Kajeepeta et al. showed in 2020 that a within-county increase in jail incarceration rates from the first to second quartile were associated with a 2.5% increase in mortality rates (adjusted risk ratio [aRR] = 1.03; 95% confidence interval [CI] = 1.02, 1.03) (Kajeepeta et al., 2020). Similarly, Weidner and Schultz (2019) showed that each 10-point increase in the predicted rate of incarceration at the county-level was associated with an 8.23-year increase in the county’s years of potential life lost (Weidner & Schultz, 2019).

**Fig. 1:**
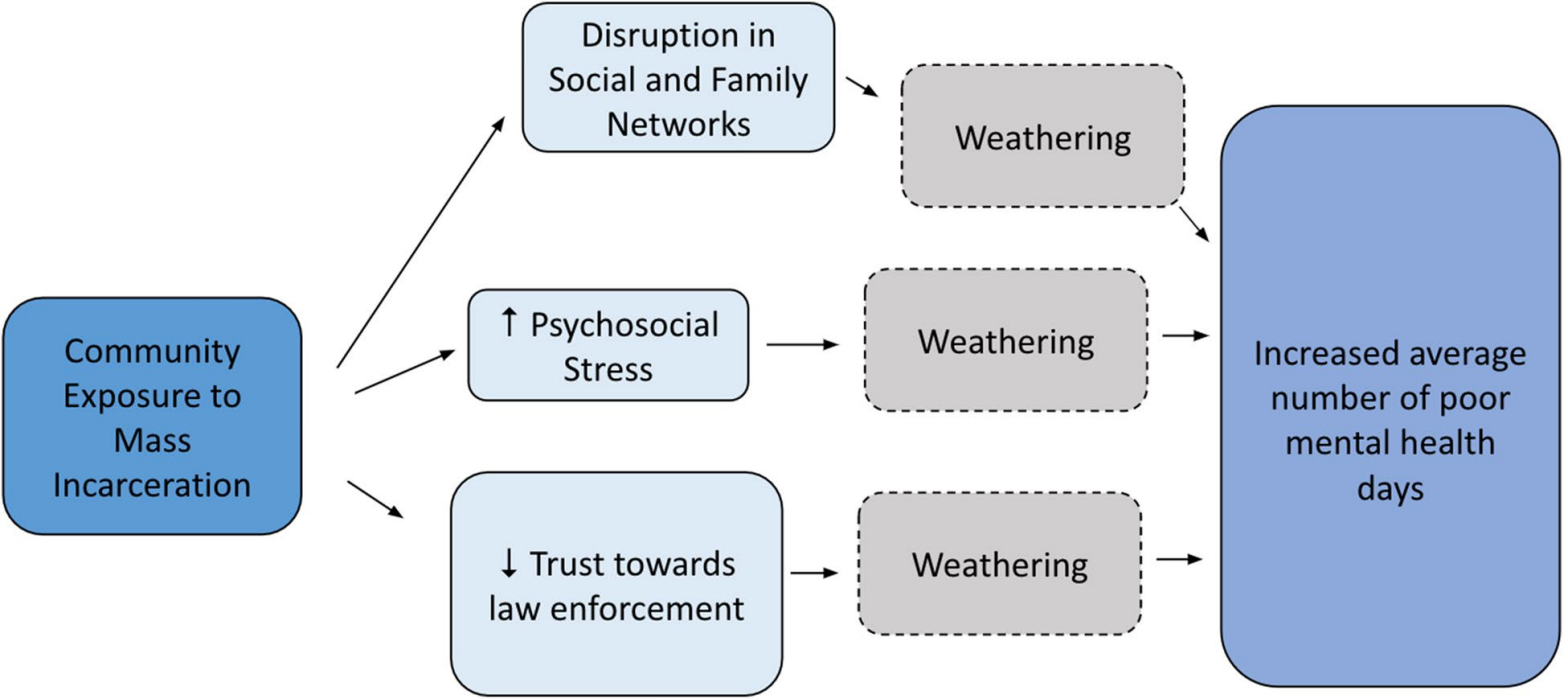
Conceptual Model

In our study, we were interested in exploring the spillover mental health consequences of county jail incarceration rates. Previous research suggests that because of the effect of low socioeconomic status, chronic stress, and social isolation on health, the incarceration of a family member may contribute to a new type of *weathering* (Lee et al., 2014). The weathering hypothesis asserts that Black people are burdened by an early physiological deterioration because of the cumulative impact of repeated exposures to social or economic adversity and political marginalization (Geronimus et al., 2006). While quantifying weathering is beyond the scope of this study, allostatic load is a measurement that may be well-suited for evaluating weathering, and as such, is necessary to include in the conceptual model (Duru et al., 2012). Allostatic load is the physiological burden that is imposed specifically by stress (Duru et al., 2012). As such, mental health is important to explore within the context of incarceration, allostatic load, and weathering. In the conceptual model, it is proposed that community exposure to mass incarceration leads to a disruption in social and family networks, an increase in psychosocial stress which induces an allostatic load response, and a decrease in trust towards law enforcement. These experiences in turn contribute to weathering and an increase in the average number of poor health mental days as reflected in Fig. 1. As such, exposure to mass incarceration is a community risk factor that disproportionately impacts certain populations and may facilitate weathering by serving as an ecologic stressor that adversely affects mental health.

The mental health spillover effects of local rates of incarceration are less known at the county level, leaving a gap in the knowledge about the acute and potentially chronic implications for people who are directly and indirectly affected by the actions of the carceral state. Given the connection between mental and physiological health, this study aims to quantify another important consequence of mass incarceration that may adversely affect the health of the nation and widen health disparities (Geronimus et al., 2006).

## Methods

### Level of analysis

Counties were used as the unit of analysis in this study to assess how local policies and practices that drive mass incarceration affect the health of the surrounding nonincarcerated population. Given people are usually confined in the county where the offense is committed, counties are an appropriate geographic boundary to determine the adverse impact local rates of incarceration have on the communities they are proximal to (A guide to the criminal justice system | CriminalJustice.com, n.d.). Additionally, county-level groupings are the smallest level of analysis that incarceration can be explored through since the jurisdictional operation of jails and courts is at the county level. Hence, county-level variability can provide possible explanations for the high rates of incarceration seen across the United States (GitHub - vera-institute/incarceration-trends: Incarceration Trends Dataset and Documentation, n.d.-a).

### Data

#### Exposure

The jail population is the basis for county incarceration counts in this study. The annual average daily jail population was extracted from the Vera Institute’s *Incarceration Trends 2.2* county-based file (GitHub - vera-institute/incarceration-trends: Incarceration Trends Dataset and Documentation, n.d.-b). Since most counties have their own jail jurisdiction, the Vera Institute aggregates individual jails to their jail jurisdiction, and then to their county (Kang-Brown et al., 2020). The county population at-risk, used for the calculation of jail incarceration rates, was considered to be those between 15 and 64 years of age, given that the age groups < 15 and > 64 years have a low incarceration risk (Kang-Brown et al., 2020).

#### Outcome

County-level age-adjusted mean number of reported number of poor mental health days within the past 30 days was extracted from the Robert Wood Johnson County Health Rankings and Roadmaps tool, which compiles data collected by the Behavioral Risk Factor Surveillance System (BRFSS) from the Centers for Disease Control and Prevention (Poor mental health days | County Health Rankings and Roadmaps, n.d.).

#### Covariates

The percentage of each county that self-identified as Black aged 15 to 64 years in 2018 was extracted from the Vera Institute’s *Incarceration Trends 2.2* county-based file (GitHub - vera-institute/incarceration-trends: Incarceration Trends Dataset and Documentation, n.d.-b). The percentage of males in the county aged 15 to 64 was initially considered as a potential covariate given that men are significantly more likely to be incarcerated than women (Criminal justice facts | The Sentencing Project, n.d.). However, this variable was removed due to multicollinearity with the other covariates in the model (Variance Inflation Factor: > 2400).

Elevated levels of violent crime are associated with higher rates of incarceration as well as compromise physical safety and psychological well-being (Dhondt, 2012; Violent crime rate | County Health Rankings and Roadmaps, n.d.). The county-level violent crime rate for 2014–16 was sourced from the Robert Wood Johnson County Health Rankings and Roadmaps tool; law enforcement agencies at the local, state and federal levels voluntarily submit violent crime data (by hierarchy of the most serious offense) by location of occurrence to the UCR program (Uniform Crime Reporting (UCR) Program — FBI, n.d.).

**Fig. 2:**
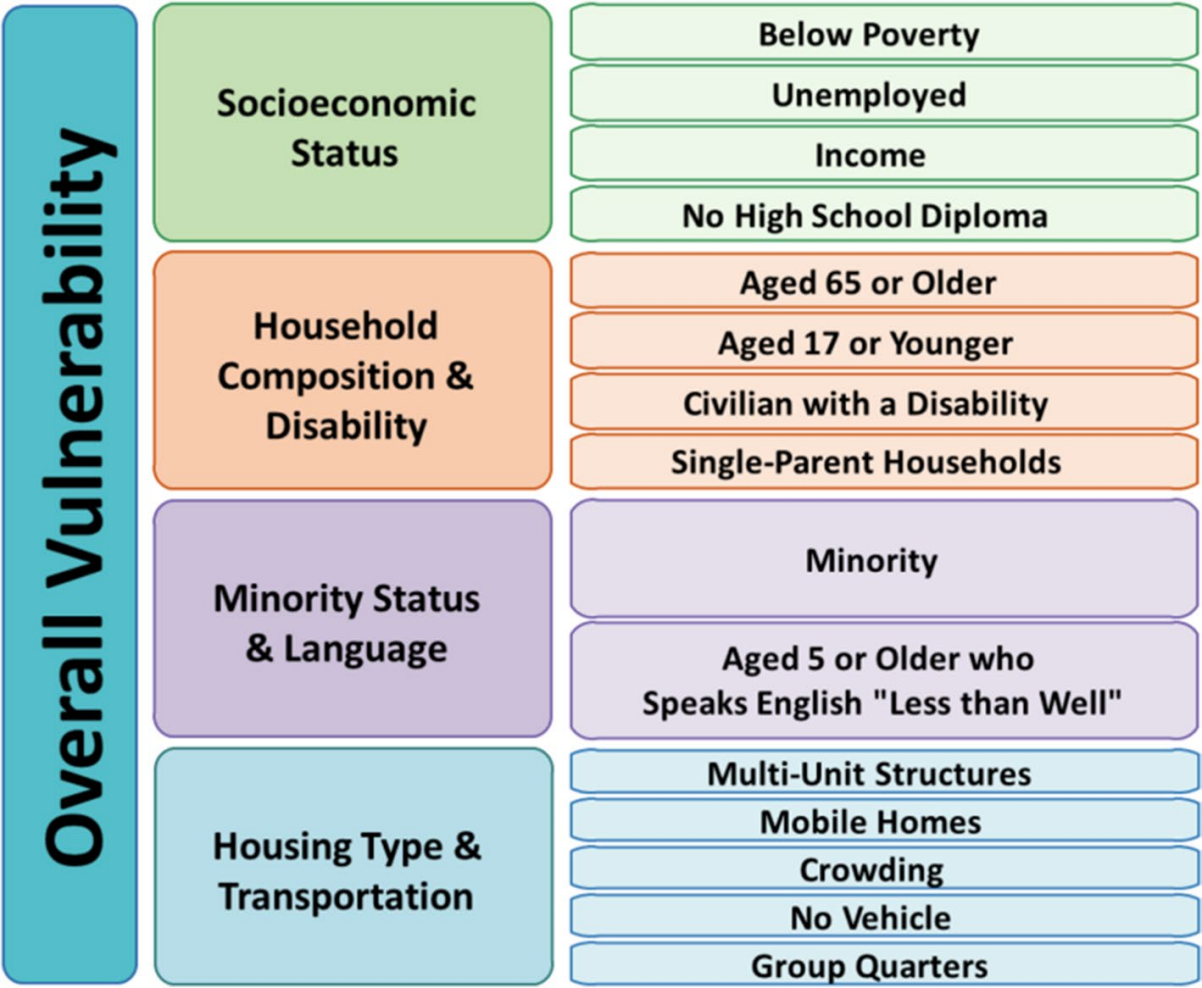
Social Vulnerability Index

The final covariate is the Centers for Disease Control and Prevention’s Social Vulnerability Index (SVI) (The 2018 CDC SVI Data and Documentation file, n.d.). Counties are ranked for the entire United States against each other, with percentile ranking values ranging from 0 (low vulnerability) to 1 (high vulnerability) (CDC SVI Documentation 2018 | Place and Health | ATSDR, n.d.). Figure 2 shows the 15 social factors that are evaluated. SVI is included as a covariate in this model because many of the risk factors evaluated are associated with higher rates of incarceration and poor mental health days.

### Data analysis

Descriptive statistics were used to show the demographic trends in the full sample of counties, along with a stratified table showing demographic trends within tertiles of level of mass incarceration. A negative binomial regression model with a cluster variable for state and the total population aged 15 to 64 as the offset was used to examine the association between the age-adjusted reported number of poor mental health days in the past 30 days and incarceration rate per 100 at the county level. A negative binomial regression was utilized to model over-dispersed count data (Negative binomial regression | Stata Data Analysis Examples, n.d.). There was no evidence of correlation between the SVI and the violent crime rate (Pearson *R*^2^: − 0.021). In addition, there was no apparent multicollinearity in the model, as confirmed by a Variance Inflation Factor (VIF) of 3.09. All analyses were done using R Version 3.6.2 and Stata Version 16.

## Results

**Fig. 3:**
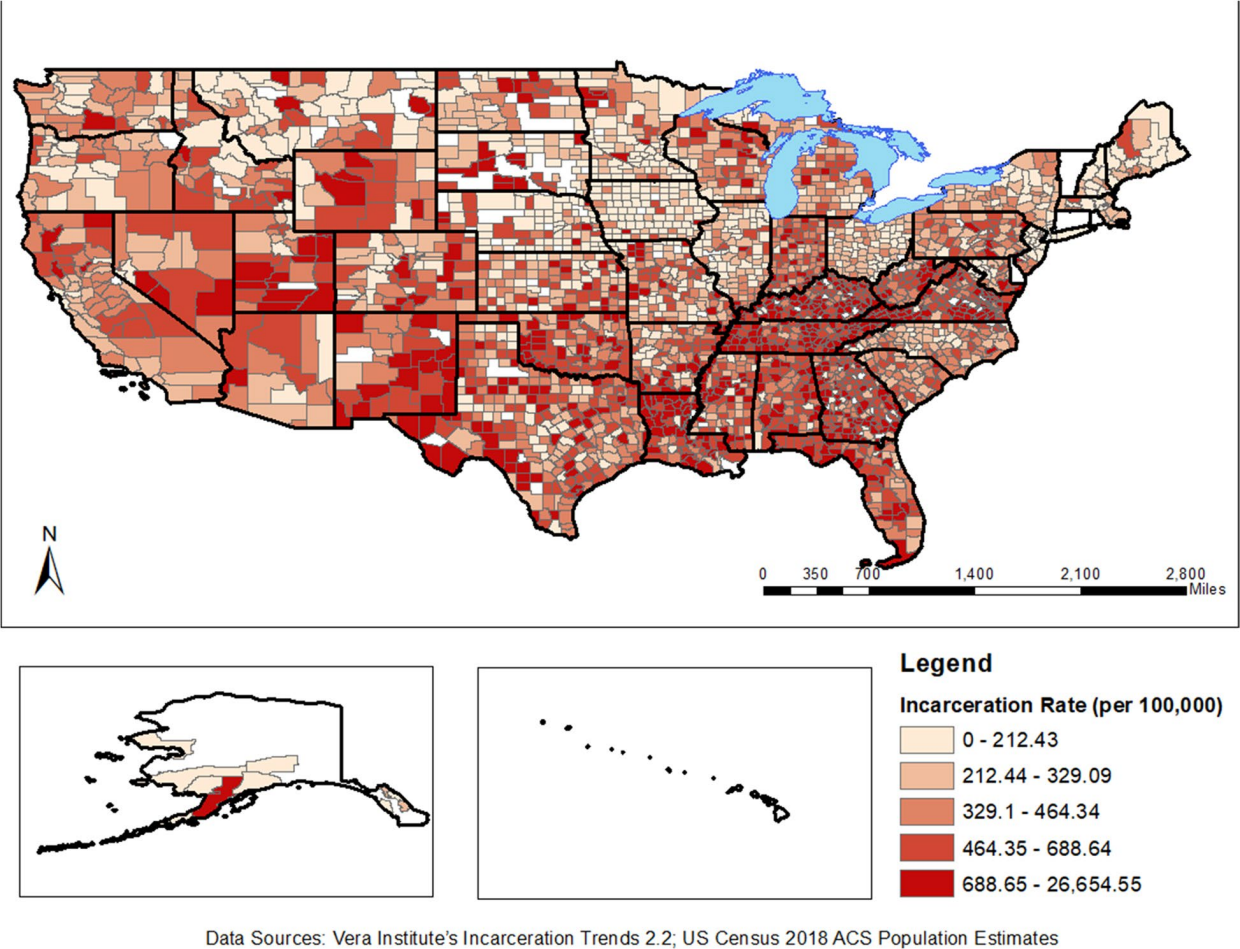
Map of County Level Incarceration Rates 2018

A total of 2823 counties with complete data were included in this analysis (Table 1). The mean incarceration rate for the sample was 604.71 per 100,000 (Standard Deviation [SD] = 1329.77); the mean number of poor mental health days within a 30-day period was 3.95 ([SD] = .60). The mean SVI was .51 ([SD] = .28), indicating a moderate to high level of vulnerability in the sample (CDC SVI frequently asked questions (FAQ) | Place and Health | ATSDR, n.d.). The mean Black population aged 15–64 was 9919.27 ([SD] = 44,882.45), and the mean number of violent crimes was 391.67 ([SD] = 1656.84). While many states such as California, have pockets of high rates of incarceration, overall county-level rates of incarceration are more heavily concentrated in the southeastern United States (Fig. 3).

**Table 1:** Descriptive statistics for the sample

	Incarceration Rate (per 100,000) ***N*** = number of counties							
	Overall		Tertile 1 (*n* = 941)		Tertile 2 (*n* = 941)		Tertile 3 (*n* = 941)	
	***M***	***SD***	***M***	***SD***	***M***	***SD***	***M***	***SD***
Mass Incarceration Rate	604.71	1329.77	188.99	67.97	399.94	66.70	1225.19	2167.75
Mentally Unhealthy Days	3.95	0.60	3.65	0.57	4.02	0.54	4.18	0.56
SVI Index	0.51	0.28	0.36	0.26	0.53	0.27	0.63	0.26
Proportion Population Aged 15 to 64 years Self-Identified as Black	9,919	44,882	14,110	66,972	10,034	32,540	5,613	21,619
No. of Violent Crimes	392	1657	559	2479	430	1205	187	756

Tertile Breakdown by Incarceration Rate:

• Final sample size: *n* = 2823 counties

• Tertile 1 (*n* = 941 counties): [0.00–294.81]

• Tertile 2 (*n* = 941 counties): (294.81–529.07]

• Tertile 3 (*1* = 941 counties): (529.07–26,654.55]

Two county-level covariates demonstrated a dose-response relationship with changes in jail incarceration rates (Table 1). A within-county change in jail incarceration rate from the first to second tertile was associated with a 10.14% increase in the average number of reported poor mental days. The change from the second tertile to the third tertile resulted in a 3.98% increase, and a change between the first and third tertile in jail incarceration rate resulted in a 14.52% increase in the average number of reported poor mental health days. The SVI increased by 75% with a county change in jail incarceration rate from the first to third tertile. Interestingly, between the first and third tertile of incarceration rates, the percentage of the county that was Black aged 15–64 declined by 60%. Similarly, the number of violent crimes decreased by 67% between the first and third tertiles of jail incarceration rate between the first and third tertile.

For every 1% increase in the county incarceration rate, there was a statistically significant 15% increase in the number of poor mental health days (*P <* .001*,* adjusted IRR: 1.15, 95% CI: 1.08, 1.21) (Table 2). The remaining covariates in the model had effect sizes of ~ 1 indicating essentially no association with number of reported poor mental health days. Because SVI includes a racial and/or ethnic identity as an indicator of vulnerability, we did a sensitivity analysis excluding proportion of the county that is Black aged 15–64; the results were nearly identical (data not shown). The purpose of conducting the sensitivity analysis was to ensure we were not over-controlling for this construct by having it in the index as well as having it as a separate covariate. By confirming the results were similar, more credence was given to the appropriateness of our primary model.

**Table 2:** Adjusted negative binomial model

Indicator	Incidence Rate Ratio (IRR)	95% Confidence Interval
Mass Incarceration (per 100 population)	1.15	1.08, 1.21
Proportion Population Ages 15 to 64 years Self-Identified as Black	1.00	0.99, 1.00
SVI Index	0.95	0.60, 1.49
Violent Crime Rate	1.00	0.99, 1.00

• Poor Mental Health Days *(mean days/month*) & Incarceration Rate *(per 100)*

• Covariates: SVI Index, Proportion Black, Proportion Male, # of Violent Crimes

## Discussion

Our results show that high local rates of incarceration are positively associated with the number of reported poor mental health days at the county-level. Our findings highlight another spillover consequence of mass incarceration on communities. The burden of mass incarceration and the impact on mental health is present in all counties, but counties in the 14 states in the South are disproportionately impacted due to higher regional rates of incarceration. This regional concentration is particularly concerning given the majority of the United States’ Black population lives in the South (56%), and it gives credence to how racialized and burdensome mass incarceration is as an extension of slavery and Jim Crow (Facts about the U.S. Black population | Pew Research Center, n.d.). Our study aligns with previous studies that have examined associations between incarceration and poor mental health at the individual-level; we extend this finding to elucidate the mental health spillover effects that burden communities with high rates of incarceration (Hatzenbuehler et al., 2015; Sugie & Turney, 2017).

A change in jail incarceration rate between the first and third tertile was associated with a 75% increase in the average SVI and decrease in the number of violent crimes (67%) and the percentage of the county that is Black aged 15–64 (60%). These findings suggest that the communities most burdened by high rates of incarceration at the county level do not have higher levels of violent crime or a greater percentage of residents that are categorized as racially Black. In tertile 1, where the incarceration rate was the lowest, the number of violent crimes were actually the highest. These findings are unsurprising; contrary to popular opinion, it is known that higher rates of incarceration do not influence violent crime rates (Stemen, 2017). Additionally, nearly 75% of people who were detained pretrial and sentenced are in jail for nonviolent offenses (incarcerations-front-door-infographic.jpg (1651×1275), n.d.). Our data also show that counties that have the highest rates of incarceration have a smaller proportion of Black residents than counties with the lowest incarceration rates. This is consistent with what is known about the disproportionate rates of incarceration for Black people in the United States relative to their population size (Incarceration’s front door | Vera Institute, n.d.). In counties with a smaller Black population, Black people may not comprise most of the population that is confined in local jails, but they are likely overrepresented relative to their proportion of the county’s population. Furthermore, the history of policing in the United States has largely been critiqued as an extension of slavery and Jim Crow; as such, representing a smaller percentage of the county’s population may correlate with an increased risk of confinement for Black residents (Alexander, 2012).

The only additional county-level variable that increased with changing incarceration rates was the SVI index. The 75% increase in the average SVI score between the first and third tertiles indicates that there are moderate levels of vulnerability in the counties most impacted by high jail incarceration rates. A mean SVI of 0.51 gives credence to a moderate percentage of the county scoring low on the socioeconomic status, household composition and disability, racial and/or ethnic identity and language, and housing type and transportation social vulnerability indicators. Hence, the residents in the counties most burdened by high rates of jail incarceration are already socially vulnerable, and thus, more susceptible to the deleterious effects of mass incarceration.

There are several limitations with this study. First, our study utilizes a cross-sectional design, and thus no inferences regarding a causal relationship between incarceration rates and reported poor mental health days can be drawn. However, while a cross-sectional study design will not establish a temporal relationship between the exposure and health outcome in the population, it can be very suggestive of how high incarceration rates can affect mental health in communities. This also study utilized single-year variables; future studies could look at trends over time to demonstrate patterns across different periods of time. Additionally, while the results are generalizable to most counties in the United States, a small number of counties (*n* = 319) were excluded due to being incomplete cases. The outcome variable did not have data that would allow stratification by race or gender. Given the racial disparities that exist in incarceration rates, this limitation prevents an important association from being examined. Lastly, this is an ecological study utilizing county-level averages that may not hold true for individuals. Future studies should examine the association between local rates of incarceration and mental health in cohort studies.

Mass incarceration is associated with poor mental health and well-being in the broader population given that spillover effects extend beyond those directly affected. In communities that are disproportionately burdened by high rates of local incarceration, the risk of widening existing health disparities through weathering persists. The system of policing and incarceration in the United States is overdue for restructuring beyond the public health implications that have been presented here, as several studies have demonstrated how harmful high rates of incarceration are to the community’s health (Dyer et al., 2019; Kajeepeta et al., 2021, 2020; Weidner & Schultz, 2019).

Given most justice-impacted persons in the United States are not being held for allegations that constitute serious offenses, the resources that are invested in confining and policing vulnerable communities would be better utilized improving the social conditions of county residents (Mass incarceration: The whole pie 2020 | Prison Policy Initiative, n.d.). Confinement for any length of time is harmful to entire communities and has a negligible impact on recidivism as the conditions that lead to an arrest are unlikely to change drastically within the short period a person is jailed for a low-level offense. Future practice-based and research interventions should focus on identifying which county-level stressors and conditions increase the risk of incarceration and the number of poor mental health days in localities with high rates of incarceration. These explorations would elucidate solutions to dismantle a problematic criminal justice system that punishes the nation’s most vulnerable residents and compromises the mental health of entire communities.

## Data Availability

All data generated or analyzed during this study are included in this published article [and its supplementary information files].
